# Reduced myotube diameter, atrophic signalling and elevated oxidative stress in cultured satellite cells from COPD patients

**DOI:** 10.1111/jcmm.12390

**Published:** 2014-10-22

**Authors:** Pascal Pomiès, Julie Rodriguez, Marine Blaquière, Sami Sedraoui, Fares Gouzi, Gilles Carnac, Dalila Laoudj-Chenivesse, Jacques Mercier, Christian Préfaut, Maurice Hayot

**Affiliations:** a INSERM U-1046, University Montpellier I, University Montpellier IIMontpellier, France; b Department of Clinical Physiology, CHRU MontpellierMontpellier, France

**Keywords:** COPD, muscle dysfunction, cellular model, atrophy, oxidative stress, satellite cells

## Abstract

The mechanisms leading to skeletal limb muscle dysfunction in chronic obstructive pulmonary disease (COPD) have not been fully elucidated. Exhausted muscle regenerative capacity of satellite cells has been evocated, but the capacity of satellite cells to proliferate and differentiate properly remains unknown. Our objectives were to compare the characteristics of satellite cells derived from COPD patients and healthy individuals, in terms of proliferative and differentiation capacities, morphological phenotype and atrophy/hypertrophy signalling, and oxidative stress status. Therefore, we purified and cultivated satellite cells from progressively frozen *vastus lateralis* biopsies of eight COPD patients and eight healthy individuals. We examined proliferation parameters, differentiation capacities, myotube diameter, expression of atrophy/hypertrophy markers, oxidative stress damages, antioxidant enzyme expression and cell susceptibility to H_2_O_2_ in cultured myoblasts and/or myotubes. Proliferation characteristics and commitment to terminal differentiation were similar in COPD patients and healthy individuals, despite impaired fusion capacities of COPD myotubes. Myotube diameter was smaller in COPD patients (*P* = 0.015), and was associated with a higher expression of myostatin (myoblasts: *P* = 0.083; myotubes: *P* = 0.050) and atrogin-1 (myoblasts: *P* = 0.050), and a decreased phospho-AKT/AKT ratio (myoblasts: *P* = 0.022). Protein carbonylation (myoblasts: *P* = 0.028; myotubes: *P* = 0.002) and lipid peroxidation (myotubes: *P* = 0.065) were higher in COPD cells, and COPD myoblasts were significantly more susceptible to oxidative stress. Thus, cultured satellite cells from COPD patients display characteristics of morphology, atrophic signalling and oxidative stress similar to those described in *in vivo* COPD skeletal limb muscles. We have therefore demonstrated that muscle alteration in COPD can be studied by classical *in vitro* cellular models.

## Introduction

Dysfunction and atrophy of the skeletal limb muscles are now recognized as important extrapulmonary manifestations of chronic obstructive pulmonary disease (COPD), contributing to impaired patient exercise tolerance, worsened quality of life and reduced survival [1–3]. Furthermore, the altered muscle strength and endurance, and the muscle atrophy, are likely due to a combination of different mechanisms, with oxidative stress being one of the most important [4–9].

An impaired capacity for muscle regeneration has also been hypothesized to explain COPD muscle atrophy [10,11]. In addition, skeletal muscle repair mechanisms seem to be altered in COPD patients showing abnormal muscle structure [8]. As satellite cells are the primary contributors to muscle tissue homoeostasis, muscle regeneration during exercise and injury, and muscle repair over the long-term [12], several groups have compared their abundance in the skeletal muscles of COPD patients and healthy individuals, and they consistently found no difference [11,13,14]. However, the number of satellite cells provides no information on proliferation and differentiation capacities or redox status, therefore, the hypothesis of a compromised maintenance of muscle mass and exhausted muscle regenerative capacity of satellite cells [11] has to be assessed. Indeed, the intrinsic capacity of satellite cells to replicate and adopt myogenic development in COPD remains unknown [11].

Primary human satellite cell culture is now a well-developed approach. It has been widely used in studies of myogenesis, muscle regenerative capacity, myotube morphology alterations, signalling pathways, and the role of oxidative stress under physiological and pathological conditions, this last including both pathologies of genetic origin, like muscle dystrophy [15,16], and acquired muscle dysfunction, like type 2 diabetes and insulin resistance [17,18]. Interestingly, the myotubes obtained from satellite cell culture in non-genetic diseases conserve some of the molecular and morphological characteristics seen *in vivo* in patient muscles, and they may thus be a useful model for studying muscle dysfunction mechanisms [17,18].

The aim of this study was thus to determine whether cultured satellite cells derived from skeletal limb muscles of COPD patients are altered in terms of proliferative and differentiation capacities, morphological phenotype and atrophy/hypertrophy signalling, and redox status in comparison with cells from healthy individuals.

## Materials and methods

### Study population

Sedentary healthy individuals were recruited on the basis of the following criteria: age from 57 to 67.5 years, no disease and less than 150 min. of moderate-to-vigorous physical activity per week. COPD patients were defined on the basis of the following criteria: dyspnea, and/or chronic cough or sputum production, and/or history of exposure to risk factors for the disease, with the diagnosis confirmed by spirometry (post-bronchodilatator FEV_1_/FVC<70%; FEV_1_: forced expiratory volume in 1 sec.; FVC: forced vital capacity) [19]. Exclusion criteria were: other respiratory diagnosis, decompensated co-morbidity, and exacerbation in the last 2 months. Functional tests are detailed in the Data S1.

### Muscle biopsy procedures and conservation

Muscle biopsies were performed in the *vastus lateralis* of the quadriceps using the usual methodology [20]. One piece of the fresh biopsy was flash frozen in a pre-cooled beaker of isopentane placed in liquid nitrogen, to avoid distortion of the tissue, and lastly conserved at −80°C. Cryosections of this biopsy specimen served to assess muscle fibre cross-sectional area (CSA) by immunohistochemistry, using an anti-dystrophin antibody. Another piece of the fresh biopsy was placed in a cryogenic tube and was then progressively frozen to −80°C for 24 hrs using a Mr. Frosty freezing container (Nalgene Fisher Scientific, Pittsburgh, PA, USA), to preserve cell integrity. The cryogenic tube was then stored in liquid nitrogen until use for myoblast isolation.

### Myoblast isolation and purification

Small explants from progressively frozen biopsies conserved in a cryogenic tube were placed in a 35-mm collagen-coated Petri dish, covered with a thin layer of 6 mg/ml Matrigel (BD Matrigel Matrix from BD Biosciences, Franklin Lakes, NJ, USA) and DMEM (Sigma-Aldrich, St. Louis, MO, USA) supplemented with 20% foetal bovine serum (FBS; Dominique Dutscher SAS, Brumath, France), 0.5% Ultroser G (BioSepra, Cergy-Saint-Christophe, France) and 20 mM Hepes (Sigma-Aldrich), as previously described [15]. After 6–8 days of culture at 37°C in an atmosphere containing 5% CO_2_, migrant cells were harvested using dispase (BD Biosciences), and then grown in 100-mm collagen-coated Petri dishes with DMEM/20% FBS/0.5% Ultroser (proliferation medium).

Satellite cells were then purified following a 30-min. incubation with an anti-CD56 (NCAM) antibody (BD Biosciences) [21], using an immunomagnetic sorting system (Miltenyi Biotec, Bergisch Gladbach, Germany). Purified myoblasts (passage 1: P1) were then grown in a 100-mm collagen-coated Petri dish in proliferation medium. The purity of the 16 myoblast cultures (eight COPD and eight healthy individuals) was evaluated after immunostaining with an anti-desmin antibody and Hoechst 33258, followed by fluorescence microscopy (see the Data S1). Data analysis of more than 200 cells per culture showed a high and comparable purity of the myoblast cultures derived from healthy individuals and patients [99.8% (97.8–100) *versus* 99.7% (98.9–100); *P* = 0.721]. Myoblasts were always used at a passage below P4 for the experiments.

When myoblasts reached 80% confluence, myogenic differentiation was induced by changing the proliferation medium to DMEM/2% FBS (differentiation medium). Myotubes were obtained after 6 days in differentiation medium.

### Myoblast and myotube characterization, oxidative stress assessment, antibodies and reagents, quantitative polymerase chain reaction (qPCR) and primers

Full details are given in the Data S1.

### Statistical analysis

Variables were compared between COPD and control groups using the Student's *t*-test or the Mann–Whitney test to account for non-parametric data distribution, and data are presented as median (25th percentile–75th percentile), except for the H_2_O_2_-induced oxidative stress experiment (Fig. 8), where data are presented as the means ± standard errors (SEM). Statistical analyses were performed with SigmaStat. Significance is at *P* ≤ 0.05.

## Results

### Characteristics of the study groups

The clinical and functional characteristics of the study groups are presented in Table 1. The median predicted FEV_1_ value indicated severely impaired lung function and the BODE index [22] indicated moderate-to-severe COPD clinical states. Both 6-minute walking distance (6MWD) and quadriceps muscle voluntary contraction (MVC) values indicated significant exercise limitation and muscle dysfunction in the COPD group. Although the included COPD patients were not selected on a specific phenotype, our patient group reflects a COPD population with a significant impaired clinical state. Fibre CSA tended to be lower in the eight patients compared to the eight healthy individuals (Table 1; *P* = 0.14). However, our study groups were extracted from larger and gender-matched populations of COPD patients (*n* = 37, 31 males/6 females) and healthy individuals (*n* = 14, 12 males/2 females), in which the fibre CSA was significantly lower in COPD patients versus healthy individuals [4588 μm^2^ (3022–5731) *versus* 5463 μm^2^ (4630–6453); *P* = 0.026], and was close to our present working groups [Table 1; 4091 μm^2^ (3090–5178) *versus* 5671 μm^2^ (4789–6618); *P* = 0.14].

**Table 1:** Characteristics of the study groups

	Control individuals	COPD patients	*P*-value
*n*	8	8	
Gender (M/F)	7/1	7/1	
Age (years)	62.0 (57.0–67.5)	59.0 (51.0–65.0)	0.44
BODE index	–	6.5 (5.0–9.0)	–
FEV_1_/FVC (%)	75.0 (68.8–81.5)	39.0 (30.5–40.8)	0.003
FEV_1_ (% pred.)	102.0 (93.0–105.0)	31.5 (25.5–32.5)	<0.001
BMI (kg/m^2^)	25.9 (24.3–27.8)	22.4 (19.3–26.3)	0.19
FFMI (kg/m^2^)	19.4 (18.4–20.5)	17.4 (16.6–19.3)	0.28
6MWD (% pred.)	88.0 (83.5–97.0)	64.0 (48.0–74.0)	0.001
qMVC (kg)	27.7 (25.1–31.6)	14.1 (10.8–16.9)	0.035
Fibre CSA (μm^2^)	5671 (4789–6618)	4091 (3090–5178)	0.14

FEV_1_, forced expiratory volume in 1 second; FVC, forced vital capacity; BMI, body mass index; FFMI, fat-free mass index; 6MWD, 6-minute walking distance; qMVC, quadriceps muscle voluntary contraction; CSA, cross-sectional area. The BODE index takes into account the body mass index, the airflow obstruction, the functional dyspnoea and the exercise capacity [22].

### Healthy individual and COPD myoblasts have similar proliferation characteristics

**Fig. 1:**
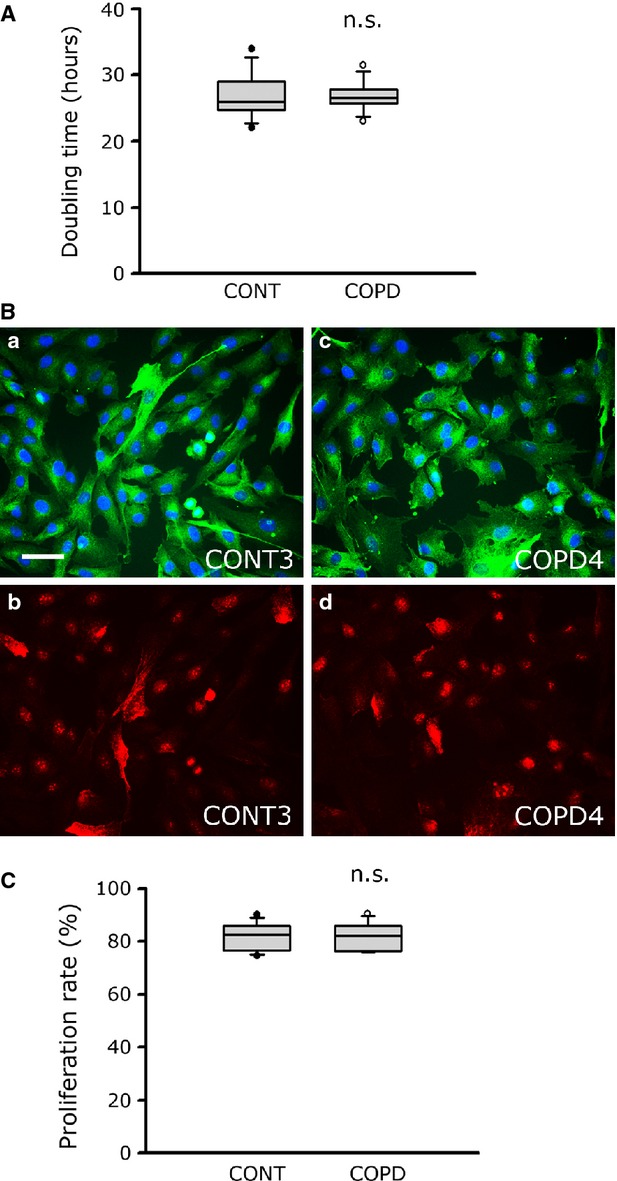
Proliferation characteristics for healthy individual and COPD cultured myoblasts. (**A**) Analysis of doubling time values of cultured myoblasts from healthy controls (CONT) and COPD patients (COPD). (**B**) Representative images of myoblasts from one control culture (CONT3) and one COPD culture (COPD4) showing fluorescence triple-labelling with an anti-desmin antibody (a, c; green), Hoechst (a, c; blue) and an anti-Ki67 antibody (b, d; red), for proliferation capacity analysis; bar = 50 μm. (**C**) Analysis of the proliferation capacity values (Ki67-positive nuclei/total nuclei) of myoblasts derived from eight controls (CONT) and eight COPD patients (COPD). (n.s.): statistically non-significant.

Myoblasts derived from healthy individuals and COPD patients were grown in proliferation medium, at the same low passage (P3), to evaluate their proliferation characteristics. The median doubling time of healthy individual and COPD myoblasts was almost identical [25.9 hrs (24.7–29.1) *versus* 26.6 hrs (25.7–27.9); *P* = 0.721], indicating similar growth capacities for healthy individual and patient cells (Fig. 1A). To confirm this result, the cell-proliferation marker, Ki67, was then used to evaluate the proliferation capacities of the healthy individual and COPD myoblast cultures. Ki67-nuclear labelling detected by fluorescence microscopy (Fig. 1B), followed by data analysis (Fig. 1C), showed comparable proliferation capacities for patients and healthy individuals [82.4% (76.5–86.0) *versus* 82.7% (76.6–86.0); *P* = 0.959].

### COPD myotubes have a normal commitment to terminal differentiation despite impaired fusion capacities

**Fig. 2:**
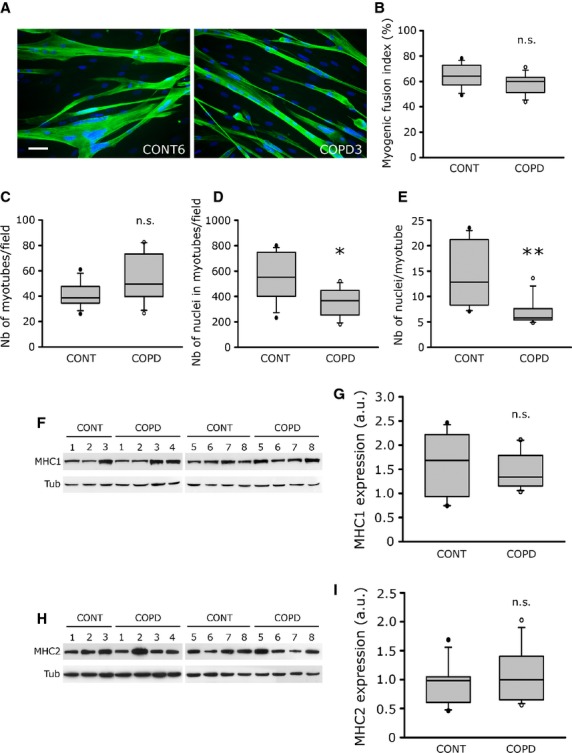
Fusion and commitment to terminal differentiation for healthy individual and COPD myotubes. (**A**) Representative images of myotubes from one control individual (CONT6) and one COPD patient (COPD3) showing fluorescence double-labelling using an anti-troponin T antibody (green) and Hoechst (blue); bar = 50 μm. (**B**) Analysis of the myogenic fusion index of cultured myotubes derived from eight control individuals (CONT) and eight COPD patients (COPD). (**C**–**E**) Analysis of the number of myotubes per field, the number of nuclei in all the myotubes per field, and the resulting number of nuclei per myotubes, respectively, in the myotube cultures from eight control individuals (CONT) and eight COPD patients (COPD). Representative Western blots showing expression levels of MHC1 (**F**) and MHC2 (**H**) in cultured myotubes derived from seven control individuals (CONT) and eight COPD patients (COPD). Tubulin is detected for loading control. Quantification of MHC1 (**G**) and MHC2 (**I**) expression relative to tubulin levels in myotubes from control individuals (CONT) and COPD patients (COPD). The mean values from the quantification of two independent Western blots are represented in arbitrary units (a.u.). (**) indicates statistical significance at *P* < 0.01; (n.s.): statistically non-significant.

After determining the myoblast characteristics, we evaluated the differentiation abilities of cultured healthy individual and COPD myoblasts placed in differentiation conditions. Figure 2A shows fluorescence microscopy images of troponin T-positive myotubes and Hoechst-labelled nuclei of representative healthy individual and COPD cultures, allowing the assessment of various fusion parameters. Analysis of the cultures indicated that the myotubes derived from healthy individuals and COPD patients had a similar myogenic fusion index [64% (57–72) *versus* 59% (51–63); *P* = 0.161; Fig. 2B], and were in an equivalent number in the cultures [38.7 (34.5–49.0) *versus* 49.5 (39.8–73.5); *P* = 0.181; Fig. 2C]. Nevertheless, the number of nuclei in myotubes per field [367 (237–461) *versus* 552 (384–749); *P* = 0.044; Fig. 2D], and the number of nuclei per myotube [5.8 (5.2–7.4) *versus* 12.8 (7.9–21.4); *P* = 0.005; Fig. 2E] are significantly reduced in COPD myotubes compared to healthy individual myotubes, suggesting that myotube fusion is impaired in COPD muscle cells. Study of the expression of myogenesis markers reveals that MyoD, Myf5 and myogenin are similarly expressed in myoblasts and myotubes from healthy individuals and COPD patients (Table 2 and Fig. S1A–G). Furthermore, the expression levels of the two late differentiation markers, myosin heavy chain 1 (MHC1) and myosin heavy chain 2 (MHC2), were assessed in healthy individual and COPD myotubes. As seen in Figure 2F–I, the expression of MHC1 [1.63 a.u. (0.93–2.21) *versus* 1.33 a.u. (1.15–1.78); *P* = 0.613] and MHC2 [0.98 a.u. (0.60–1.04) *versus* 0.99 a.u. (0.65–1.40); *P* = 0.613] were similar in myotubes derived from healthy individuals and COPD patients. Together, these data suggest that cultured COPD myotubes have a normal commitment to terminal differentiation despite impaired fusion capacities.

**Table 2:** RNA and/or protein expression of various markers of myogenesis, protein synthesis, mitochondrial biogenesis and protein breakdown, in cultured COPD myoblasts and myotubes, expressed as fold change from control cells

	RNA		Proteins	
	Myoblasts	Myotubes	Myoblasts	Myotubes
Myogenesis				
MyoD	1.24	0.81		
Myf5	1.11	1.23		
myogenin	1.25	0.69	0.95	
Protein synthesis				
IGF-1		1.00		
P-AKT/AKT			0.58*	0.90
Mito. biogenesis				
COX IV			1.11	0.58
PGC-1α	0.93	0.88		
Protein breakdown				
MuRF1	1.30	1.01	0.99	1.14
atrogin-1	1.23*	0.76		
Nedd4	0.85	0.83		
myostatin	1.45	1.67*		
FoxO1	1.41	0.75		
FoxO3	1.00	0.95		
P-ERK/ERK			1.28	0.79

Values are medians of RNA and/or protein expression in COPD cells (*n* = 8), expressed as fold change relative to expression in healthy individual cells (*n* = 8). (*) indicates statistical significance at *P* ≤ 0.05.

### COPD myotubes have a reduced diameter

**Fig. 3:**
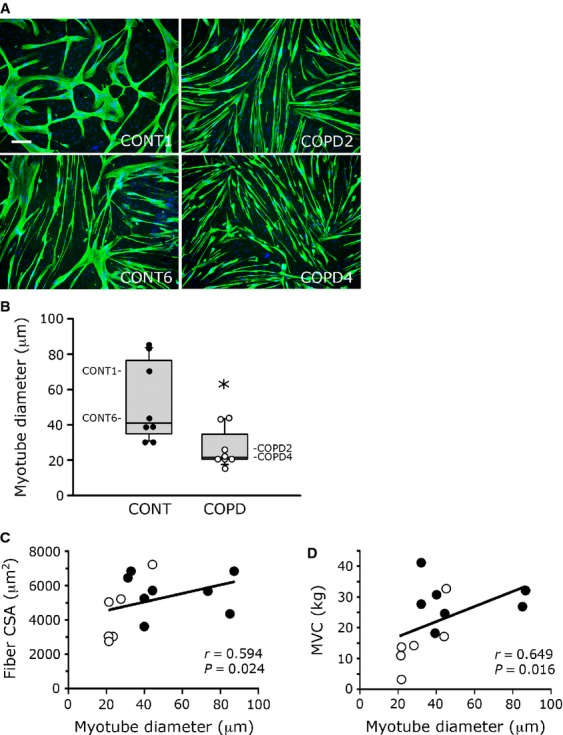
Diameter of cultured healthy individual and COPD myotubes. (**A**) Representative images of myotubes from two control cultures (CONT1, CONT6) and two COPD cultures (COPD2, COPD4) showing fluorescence double-labelling using an anti-troponin T antibody (green) and Hoechst (blue); bar = 200 μm. (**B**) Analysis of the diameter of cultured myotubes from eight controls (CONT) and eight patients (COPD). Values for each individual are shown, and values corresponding to the cultures shown in (**A**) are indicated (CONT1, CONT6, COPD2, COPD4). (*) indicates statistical significance at *P* ≤ 0.05. (**C** and **D**) Statistical analysis of healthy individuals (filled circles) and COPD patients (open circles) showing correlations between the cultured myotube diameter (Myotube diameter) and: (**C**) the quadriceps fibre cross-sectional area (Fibre CSA) and (**D**) the quadriceps maximal voluntary contraction (MVC). Data for some individuals are not represented because of unavailable values for their fibre CSA and MVC.

The diameter of the troponin T-labelled myotubes was then measured for each culture. Two representative healthy individual and two representative COPD myotube cultures observed by fluorescence microscopy are shown in Figure 3A. Analysis of the healthy individual and COPD myotube cultures (Fig. 3B) revealed that the median myotube diameter was significantly lower for COPD patients than for healthy individuals [21.6 μm (20.7–34.7) *versus* 41.1 μm (34.9–76.5); *P* = 0.015], suggesting that *in vitro* myotubes derived from COPD patients have an altered morphology. Figure 3C showed the significant correlation (*r* = 0.594; *P* = 0.024) between the myotube diameter of the *in vitro* cell cultures and the quadriceps fibre CSA of the healthy individuals and patients included in this study. We also observed a significant correlation (*r* = 0.649; *P* = 0.016) between the diameter of the cultured myotubes and the MVC values obtained for all healthy individuals and patients (Fig. 3D). Furthermore, significant correlations were also observed when only the COPD patient group was considered, between the *in vitro* myotube diameter and fibre CSA (*r* = 0.855; *P* = 0.030), as well as MVC (*r* = 0.899; *P* = 0.017; Fig. S2A and B, respectively).

### COPD myoblasts and myotubes show decreased protein synthesis and enhanced protein breakdown

**Fig. 4:**
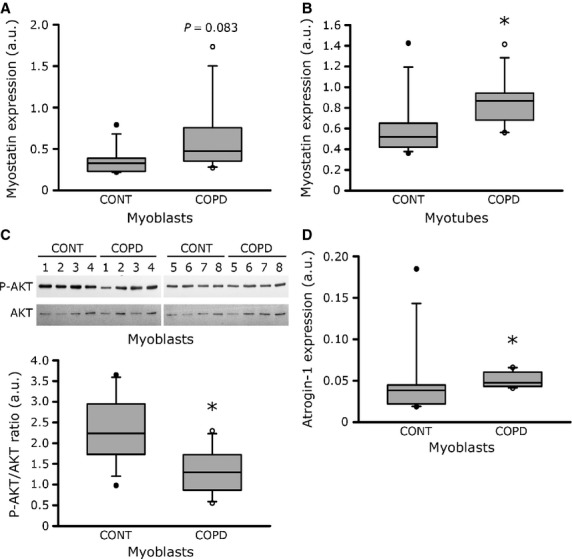
Expression levels of protein synthesis and protein breakdown markers. Myostatin mRNA expression in myoblasts (**A**) and in myotubes (**B**) from eight control individuals (CONT) and eight COPD patients (COPD). (**C**) Representative Western blots showing expression levels of phosphorylated-AKT (P-AKT) and AKT (AKT) in cultured myoblasts derived from eight control individuals (CONT) and eight COPD patients (COPD), and quantification of the P-AKT/AKT ratio. (**D**) Atrogin-1 mRNA expression levels in myoblasts from eight control individuals (CONT) and eight COPD patients (COPD). The mean values from the quantification of two independent Western blots and two independent qPCR are represented in arbitrary units (a.u.). Data are normalized to tubulin expression for protein levels, and to GAPDH expression for mRNA levels. (*) indicates statistical significance at *P* ≤ 0.05.

According to the reduced diameter of cultured COPD myotubes, we have compared the expression of various markers of the protein synthesis and protein breakdown pathways between COPD and healthy individual myoblasts and myotubes. The expression of myostatin has a tendency to be higher in COPD myoblasts than in healthy individual myoblasts [0.47 a.u. (0.33–0.86) *versus* 0.33 a.u. (0.22–0.40); *P* = 0.083; Fig. 4A], and is significantly more elevated in COPD myotubes compared to healthy individual myotubes [0.87 a.u. (0.62–0.96) *versus* 0.52 a.u. (0.42–0.65); *P* = 0.050; Fig. 4B]. Furthermore, while the P-AKT/AKT ratio is decreased [1.30 a.u. (0.78–1.90) *versus* 2.24 a.u. (1.73–3.21); *P* = 0.022; Fig. 4C], the atrogin-1 expression levels are higher [0.047 a.u. (0.043–0.063) *versus* 0.038 a.u. (0.021–0.045); *P* = 0.050; Fig. 4D] in COPD myoblasts compared to healthy individual myoblasts. These results suggest that protein synthesis is decreased and protein breakdown is enhanced in COPD muscle cells in culture. The expression of various other markers was studied, but their expression levels did not show any significant variation between COPD and healthy individual myoblasts and myotubes (Table 2).

### Oxidative stress in cultured COPD myoblasts and myotubes

**Fig. 5:**
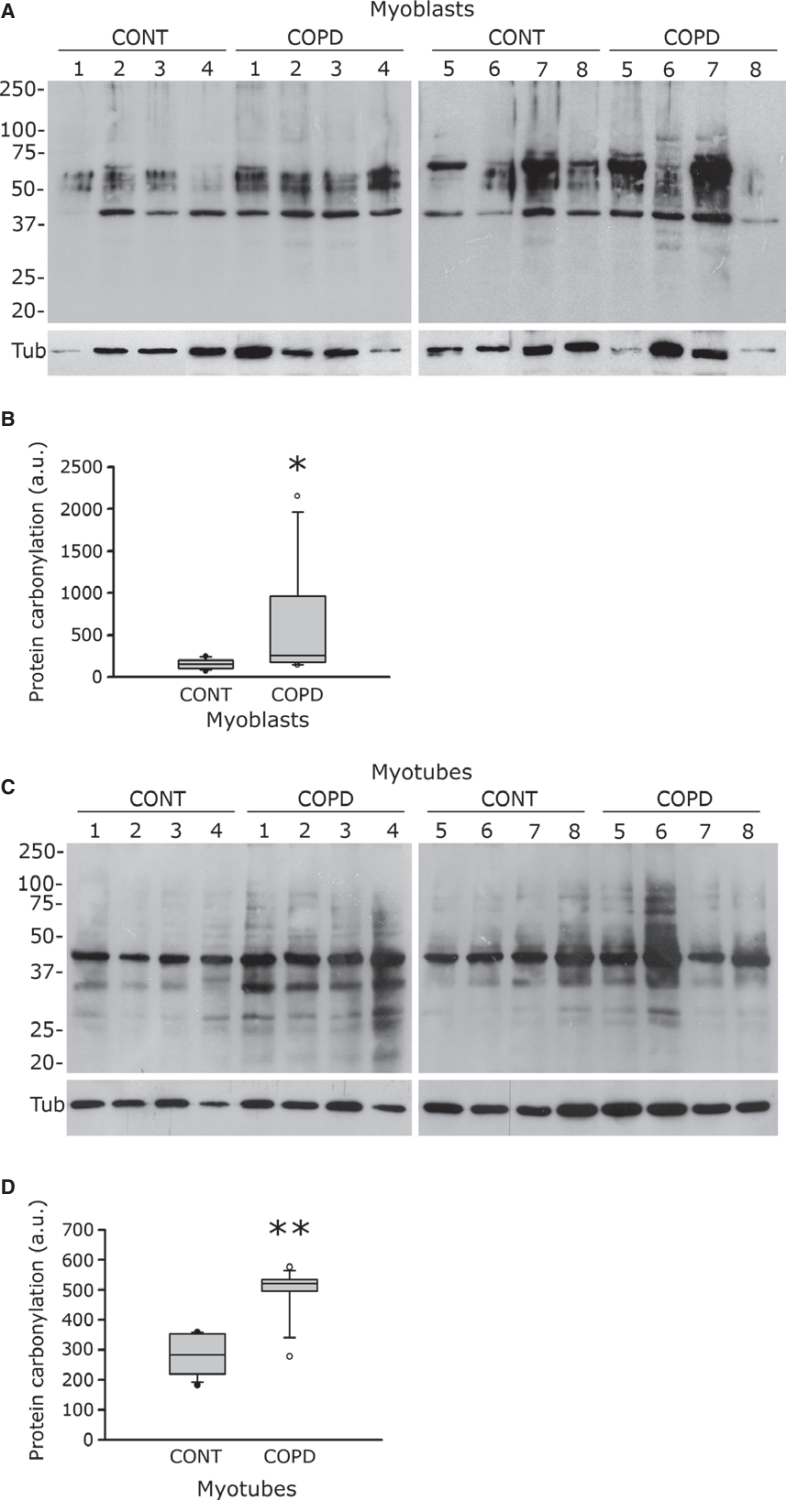
Protein carbonylation in healthy individual and COPD myoblasts and myotubes. Representative Western blots showing levels of protein carbonylation in cultured myoblasts (**A**) and myotubes (**C**) derived from control individuals (CONT1-8) and COPD patients (COPD1-8). Tubulin is also detected for loading control. Quantification of protein carbonylation relative to tubulin levels in myoblasts (**B**) and myotubes (**D**) derived from the controls (CONT) and the COPD patients (COPD). The mean values from the quantification of two independent Western blots are represented in arbitrary units (a.u.). (*) and (**) indicate statistical significance at *P* ≤ 0.05 and *P* < 0.01, respectively.

**Fig. 6:**
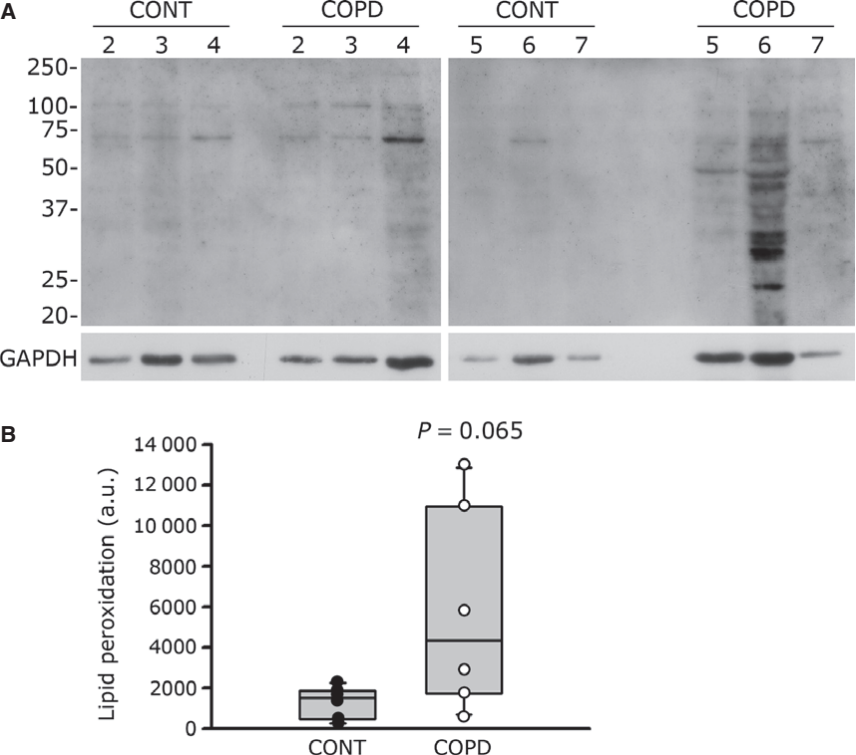
Levels of lipid peroxidation in cultured myotubes of healthy individuals and COPD patients. (**A**) Representative Western blots showing levels of HNE-modified proteins (lipid peroxidation) in cultured myotubes from controls (CONT2-7) and COPD patients (COPD2-7). GAPDH detection is used as a loading control. (**B**) Quantification of lipid peroxidation relative to GAPDH levels in myotubes from control individuals (CONT) and COPD patients (COPD). The mean values from the quantification of two independent Western blots are represented in arbitrary units (a.u.) and are shown for each individual.

Oxidative stress damage was assessed in the cultured COPD myoblasts and myotubes. Protein carbonylation was significantly more elevated in COPD than in healthy individual myoblasts [259 a.u. (182–962) *versus* 160 a.u. (103–205); *P* = 0.028; Fig. 5A and B], as well as in COPD myotubes compared with healthy individual myotubes [520 a.u. (496–534) *versus* 283 a.u. (219–353); *P* = 0.002; Fig. 5C and D]. Lipid peroxidation, as detected by the level of 4-hydroxy-2-nonenal (HNE), tended to be more elevated in COPD myotubes than in healthy individual myotubes [4347 a.u. (1745–10,946) *versus* 1512 a.u. (482–1878); *P* = 0.065; Fig. 6A and B].

**Fig. 7:**
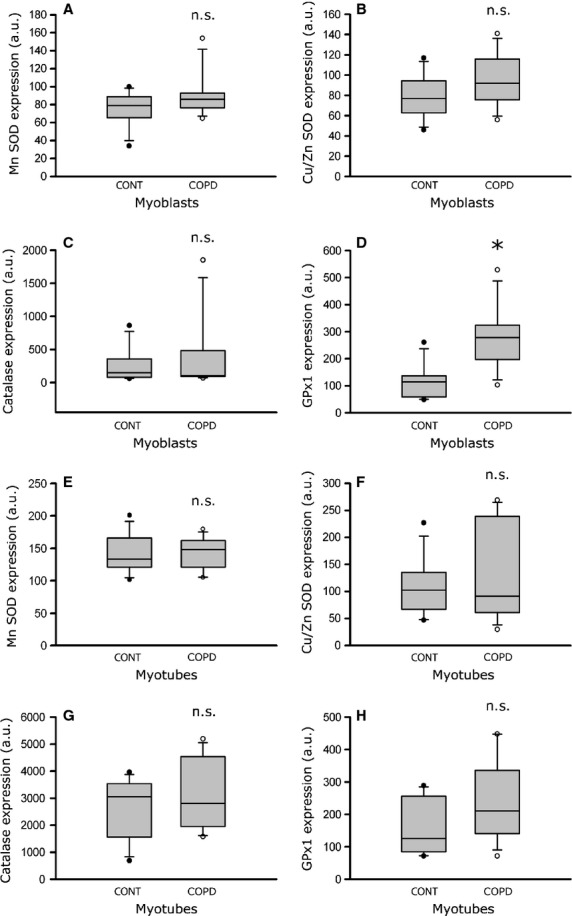
Expression levels of antioxidant enzymes in healthy individual and COPD myoblasts and myotubes. Quantification of antioxidant enzyme expression (Mn SOD, Cu/Zn SOD, catalase, GPx1) relative to tubulin expression in myoblasts (**A**–**D**) and myotubes (**E**–**H**) derived from control individuals (CONT) and COPD patients (COPD). The mean values from the quantification of three independent Western blots, for each antioxidant enzyme, are presented in arbitrary units (a.u.). (n.s.) indicates statistically non-significant, and (*) indicates statistical significance at *P* ≤ 0.05.

We next studied the expression of four major antioxidant proteins in the cultured COPD myoblasts and myotubes. As seen in Figure 7 A–D, the expression of Mn superoxide dismutase [SOD; 79.0 a.u. (65.8–88.4) *versus* 85.7 a.u. (76.8–92.4); *P* = 0.383], Cu/Zn SOD [77.0 a.u. (63.0–94.6) *versus* 92.0 a.u. (75.4–116.0); *P* = 0.318] and catalase [149.0 a.u. (82.3–356.3) *versus* 102.0 a.u. (90.7–482.4); *P* = 0.902] was similar in myoblasts derived from healthy individuals and COPD patients. Nevertheless, the glutathione peroxidase 1 (GPx1) expression level was significantly higher in COPD myoblasts than in healthy individual myoblasts [278.0 a.u. (196.8–323.5) *versus* 115.0 a.u. (59.2–137.0); *P* = 0.017]. In cultured COPD myotubes (Fig. 7 E–H), the expression level of Mn SOD [133.5 a.u. (121.5–166.0) *versus* 148.5 a.u. (121.0–162.0); *P* = 0.878], Cu/Zn SOD [102.5 a.u. (67.0–135.5) *versus* 91.5 a.u. (61.0–239.0); *P* = 0.959], catalase [3054.0 a.u. (1565.0–3534.5) *versus* 2813.0 a.u. (1951.0–4535.0); *P* = 0.463] and GPx1 [126.0 a.u. (85.0–256.3) *versus* 211.0 a.u. (141.0–336.0); *P* = 0.336] was similar to that in healthy individual myotubes.

**Fig. 8:**
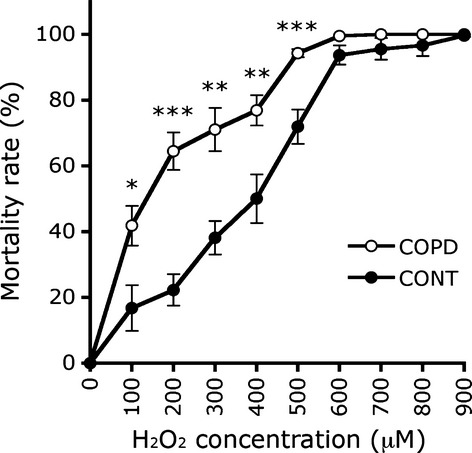
Susceptibility to H_2_O_2_-induced oxidative stress for healthy individual and COPD myoblasts. Mortality rate analysis of control (CONT; filled circles) and COPD (COPD; open circles) myoblasts grown in proliferation medium for 18 hrs with increasing concentration of H_2_O_2_. Experiments were performed for the eight healthy individual and eight COPD cultures. Data are presented as the mean ± SEM for each H_2_O_2_ concentration. (*), (**) and (***) indicate statistical significance at *P* ≤ 0.05, *P* < 0.01 and *P* < 0.001, respectively, between CONT and COPD for each H_2_O_2_ concentration.

We also examined the susceptibility of the cultured COPD myoblasts to an induced oxidative stress by exposing the cells to increases in the concentration of H_2_O_2_. Figure 8 shows that the mortality rate for the COPD myoblasts was significantly higher than for the healthy individual myoblasts at H_2_O_2_ concentrations from 100 to 500 μM, with almost 100% mortality at concentrations greater than 600 μM for both study groups. Furthermore, the H_2_O_2_ concentration necessary to produce a 50% cell death rate was 392 ± 33 μM for healthy individual myoblasts compared with 148 ± 28 μM for COPD myoblasts (*P* < 0.001).

## Discussion

The major finding of this study is that myoblasts and myotubes obtained from cultured satellite cells derived from the skeletal muscle of COPD patients are altered compared with cells from healthy individuals. Although the COPD myoblasts exhibited growth capacities similar to those of healthy individual cells and the COPD myotubes had a normal commitment to terminal differentiation, we observed that: (*i*) COPD myotubes had impaired fusion capacities, (*ii*) the cultured COPD myotubes showed significant reduced diameter compared with healthy individual myotubes, (*iii*) COPD myoblasts and myotubes showed decreased protein synthesis associated with increased protein breakdown, (*iv*) protein oxidation and lipid peroxidation were more elevated in myoblasts and myotubes from COPD patients, and (*v*) the COPD myoblasts were more susceptible to oxidative stress than healthy individual myoblasts. Together, our data indicate that *in vitro* myoblasts and/or myotubes derived from COPD patients display characteristics of reduced diameter, atrophic signalling and elevated oxidative stress similar to those described in *in vivo* skeletal limb muscles of COPD patients.

Cultured myotubes derived from human satellite cells have been shown to display morphological and biochemical characteristics similar to those of *in vivo* human skeletal muscles, under both physiological [23] and pathological conditions like the insulin resistance of type 2 diabetes [17,18]. For this reason, cultured human satellite cells have been successfully used as a cellular model to study muscle regeneration during ageing [24], the muscle biochemical characteristics in type 2 diabetes [25,26], and the susceptibility of muscle to oxidative stress and muscle differentiation in facioscapulohumeral dystrophy [15,16]. We show here that a single progressively frozen muscle biopsy from a COPD patient gave access to millions of purified myoblasts that can be expanded and that retained the capacity to differentiate into myotubes, allowing us to carry out multiple cellular and biochemical studies starting with minimal *in vivo* samples.

The myoblast and myotube cultures demonstrated that proliferation characteristics and commitment to terminal differentiation were not affected in cells derived from COPD patients (Figs 2 and Table 2). Our *in vitro* findings are therefore in accordance with some *in vivo* data showing that no major morphological abnormalities are present in COPD muscle biopsies, in terms of central nuclei, fibre splitting, regenerating fibres and apoptosis, despite the significant atrophy of muscle fibres in these patients [27]. Furthermore, muscle regenerative capacity, as reflected by the number of satellite cells per muscle fibre, is not altered in patients with COPD [13].

Studies using computed tomography have demonstrated that *in vivo* thigh muscle CSA is reduced in COPD patients [28] and that midthigh muscle CSA is a good predictor of mortality in these patients [2]. In addition, this reduced muscle CSA may explain the reduced quadriceps strength in a population of healthy individuals and COPD patients combined [28]. One of the most interesting findings of our study is the significant reduced myotube diameter observed in cultured cells derived from COPD patients (Fig. 3). Moreover, we observed a correlation between the *in vitro* myotube diameter and both *in vivo* quadriceps fibre CSA and *in vivo* muscle strength (Fig. 3). We also showed that the reduced COPD myotube diameter could result from two mechanisms. First, COPD myotubes have a reduced number of nuclei per myotubes (Fig. 2), suggesting impaired fusion capacities that would result in thinner myotubes. Secondly, we observed an increased expression of the muscle growth inhibitor myostatin and of the muscle-specific ubiquitin E3 ligase atrogin-1 (Fig. 4), showing that atrophic signalling pathways are activated in cultured COPD muscle cells. In parallel, the protein synthesis pathway is repressed in COPD cells as observed by the reduced P-AKT/AKT ratio (Fig. 4). Interestingly, it has been demonstrated that myostatin plays a central role in muscle wasting as it activates myotube atrophy through negative regulation of AKT signalling [29] and positive modulation of the atrogin-1-dependent proteasome pathway [29,30]. The *in vitro* reduced COPD myotube diameter could therefore result from a combination between impaired myoblast fusion, a mechanism that has not been evocated in the COPD literature yet and that could be a novel pathway to explore, and increased atrophic signalling, a pathway that has been reported in the limb muscles of COPD patients by several authors [31–33]. Our *in vitro* data are therefore in accordance with what is observed in COPD patients, which suggests that the cellular model could be used to study the molecular mechanisms involved in COPD muscle atrophic remodelling.

Elevated oxidative stress, as indicated by increased levels of protein carbonylation and lipid peroxidation, was observed in the cultured myoblasts and myotubes derived from COPD patients (Figs 5 and 6). Under these conditions, constant or higher expression levels of antioxidant enzymes (Fig. 7) suggest that the elevated oxidative stress in cultured COPD myoblasts and myotubes cannot be fully overcome by the antioxidant defence mechanisms present in COPD muscle cells. These data are therefore in accordance with the increased susceptibility to oxidative stress we observed in the cultured myoblasts (Fig. 8). In various studies, similar high oxidative stress has been demonstrated in human biopsies, as indicated by increased lipid/protein oxidative damage [4–6,8] and constant or higher expression levels of antioxidant enzymes [4,8] in the skeletal limb muscles of COPD patients. Furthermore, in this present work, our study groups were extracted from a larger population in which we have observed significant higher levels of protein carbonylation in the quadriceps of COPD patients (*n* = 30, 27 males/3 females) compared to healthy individuals (*n* = 24, 11 males/13 females; 143 ± 73 a.u. *versus* 106 ± 37 a.u.; *P* = 0.026). The *in vitro* reduced myotube diameter, atrophic signalling and elevated oxidative stress observed with the cellular model thus reflect the oxidative stress-induced peripheral muscle dysfunction observed *in vivo* in COPD patients.

Interestingly, in this study, the satellite cells from COPD patients conserved pathological characteristics, such as elevated intrinsic oxidative stress, even when they were taken out of their physiological context and placed in *in vitro* culture conditions. Different hypotheses can be proposed to explain this mechanism. First, a genetic defect in the satellite cells of COPD patients might be the cause, even though various single nucleotide polymorphism studies have only shown a restricted association with COPD status [34–36]. Second, mitochondria from COPD skeletal muscle show significant dysfunction associated with elevated levels of ROS [37], and a decrease in mitochondrial DNA content is observed in the skeletal muscle of COPD patients following exercise [38]. One might thus assume that mitochondrial dysfunction and elevated ROS in *in vivo* COPD muscles also affect satellite cells, which conserve their pathological characteristics when placed in *in vitro* conditions. Last, epigenetics can result in inheritable changes in cell phenotype in response to environmental factors through the methylation of DNA, and it has been recently demonstrated that ROS can modulate the expression of several genes by DNA methylation [39,40]. We can therefore speculate that gene expression may be altered *in vivo* in the muscle satellite cells of COPD patients by ROS-induced DNA methylation and that this epigenetic modulation could be transmitted to *in vitro* satellite cells. Thus, the *in vitro* cellular model developed in this study should allow us to study these different hypotheses.

In summary, we demonstrated that cultured satellite cells derived from skeletal limb muscles of COPD patients have a proliferative capacity and a commitment to terminal differentiation similar to those of cells from healthy individuals. We also showed that *in vitro* myotubes from COPD patients have a reduced diameter associated with an increased atrophic signalling, and that cultured myoblasts and myotubes from these patients display elevated oxidative stress. Thus, *in vitro* myoblasts and myotubes derived from COPD satellite cells exhibit characteristics of morphology, atrophy and oxidative stress similar to those of *in vivo* quadriceps muscles from COPD patients. We propose that this *in vitro* model provides a promising basis for research into COPD muscle alteration, which is a key component of muscle dysfunction and atrophy in patients.

## Supporting information

**Figure S1** Expression levels of myogenesis markers. MyoD mRNA expression in myoblasts (**A**) and myotubes (**B**). Myf5 mRNA expression in myoblasts (**C**) and myotubes (**D**). Myogenin mRNA expression in myoblasts (**E**) and myotubes (**F**). Data are normalized to GAPDH expression. (**G**) The mean values from the quantification of two Western blots assessing myogenin expression in cultured myoblasts at 2 days of differentiation, relative to tubulin expression. Cells are derived from eight control individuals (CONT) and eight COPD patients (COPD). Values are represented in arbitrary units (a.u.).

**Figure S2** (**A** and **B**) Statistical analysis of patients in the COPD group showing correlations between the cultured myotube diameter (Myotube diameter) and: (**A**) the quadriceps fibre cross-sectional area (Fibre CSA) and (**B**) the quadriceps maximal voluntary contraction (MVC). Data for some individuals are not presented because of unavailable values for their fibre CSA and MVC.
