# Supplementary figures and images for: Reduced myotube diameter, atrophic signalling and elevated oxidative stress in cultured satellite cells from COPD patients
Source: J Cell Mol Med. 2014 Oct 22;19(1):175–86. doi: 10.1111/jcmm.12390 (PMC4288361; doi:10.1111/jcmm.12390)

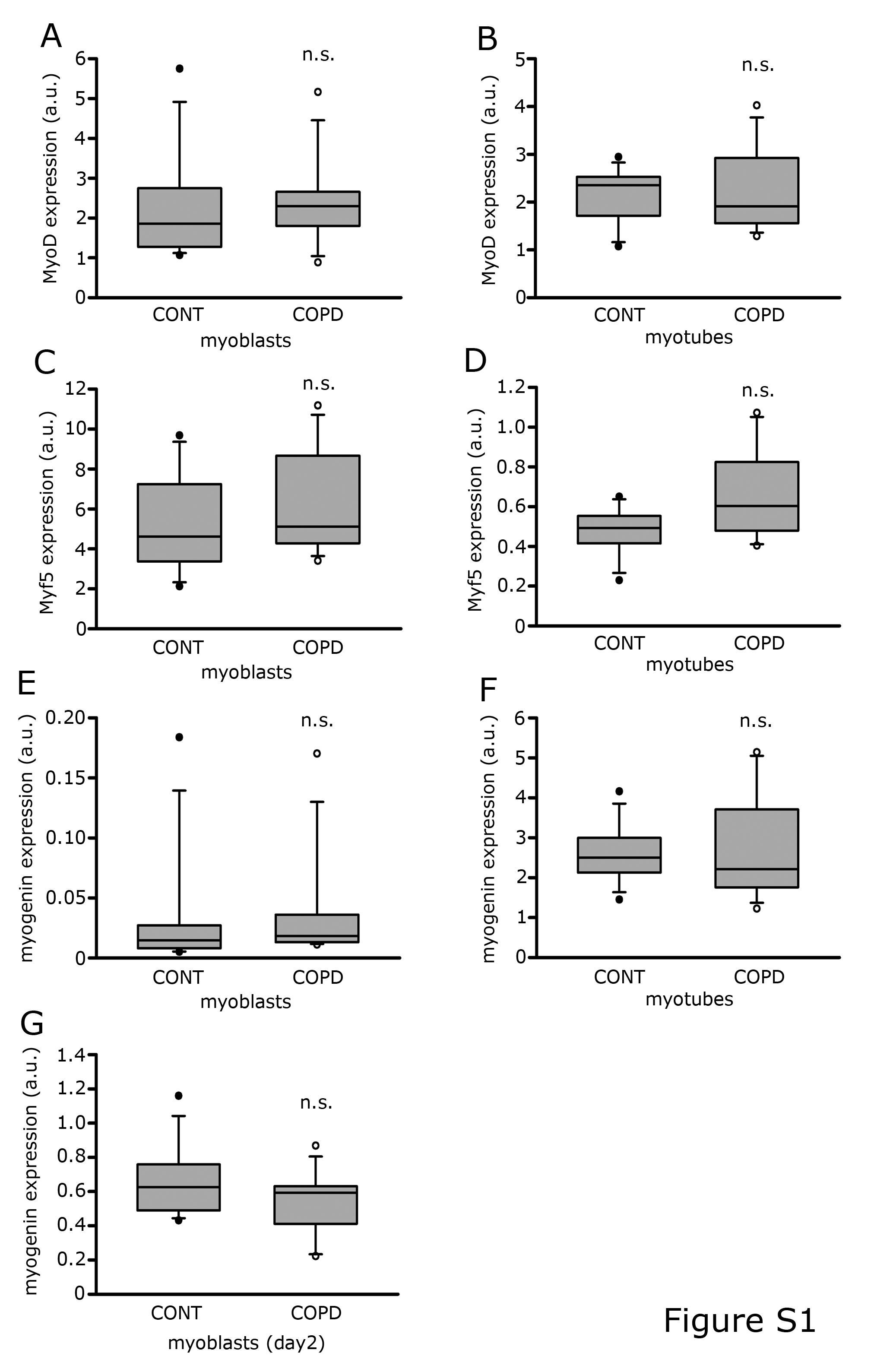

Supplement: Supplementary file 1 — Figure S1 Expression levels of myogenesis markers. MyoD mRNA expression in myoblasts (A) and myotubes (B). Myf5 mRNA expression in myoblasts (C) and myotubes (D). Myogenin mRNA expression in myoblasts (E) and myotubes (F). Data are normalized to GAPDH expression. (G) The mean values from the quantification of two Western blots assessing myogenin expression in cultured myoblasts at 2 days of differentiation, relative to tubulin expression. Cells are derived from eight control individuals (CONT) and eight COPD patients (COPD). Values are represented in arbitrary units (a.u.). [file jcmm0019-0175-sd1.tif]

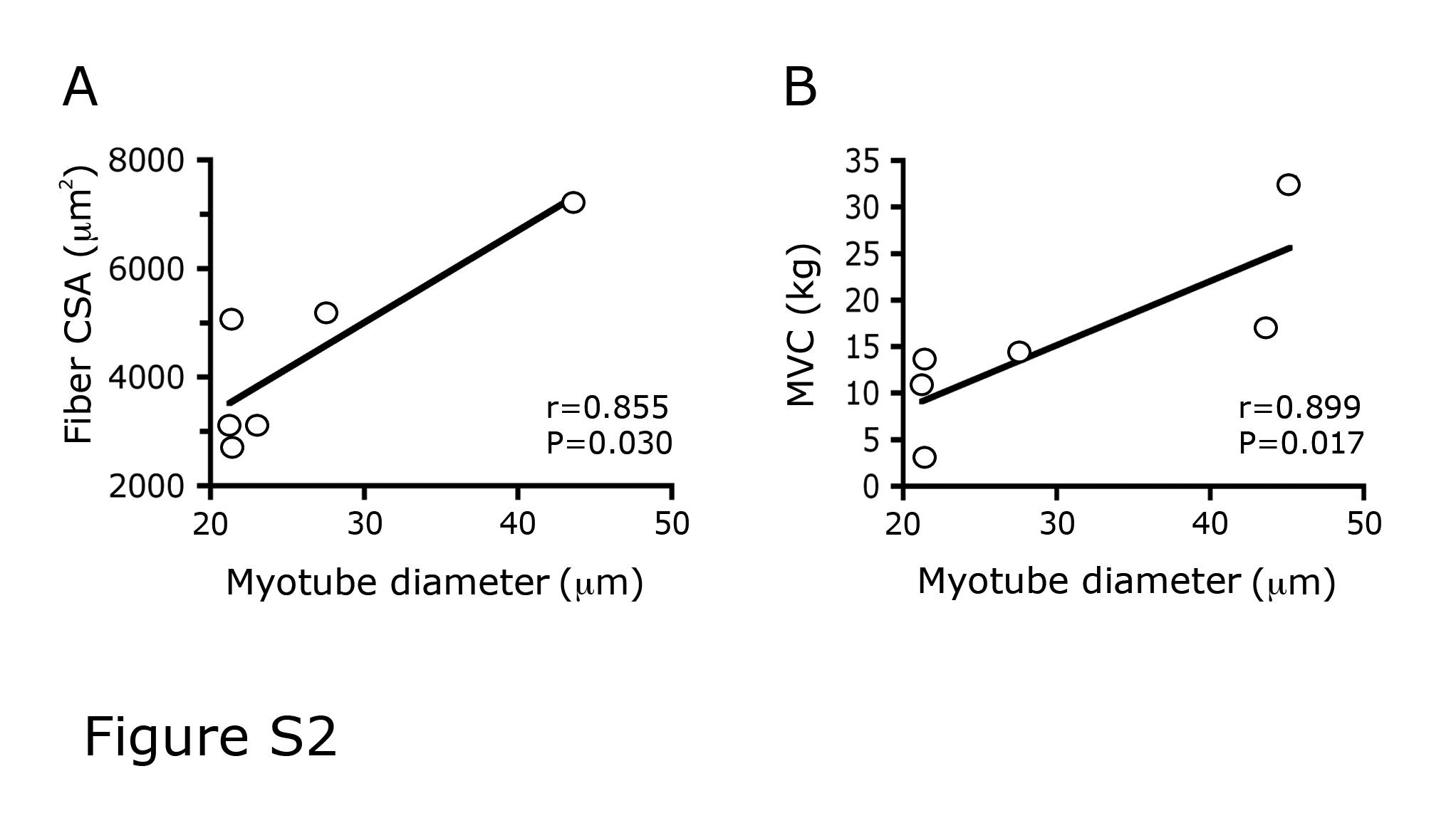

Supplement: Supplementary file 2 — Figure S2 (A and B) Statistical analysis of patients in the COPD group showing correlations between the cultured myotube diameter (Myotube diameter) and: (A) the quadriceps fibre cross-sectional area (Fibre CSA) and (B) the quadriceps maximal voluntary contraction (MVC). Data for some individuals are not presented because of unavailable values for their fibre CSA and MVC. [file jcmm0019-0175-sd2.tif]
