# Supplementary material for: Reduced myotube diameter, atrophic signalling and elevated oxidative stress in cultured satellite cells from COPD patients
Source: J Cell Mol Med. 2014 Oct 22;19(1):175–86. doi: 10.1111/jcmm.12390 (PMC4288361; doi:10.1111/jcmm.12390)
Supplement: Supplementary file 3 — Data S1 Materials and methods. [file jcmm0019-0175-sd3.doc]

**Supporting Information**

**Materials and methods**

**Research protocol**

Informed written consent was obtained from all subjects, and the research protocol was approved by the institutional ethics committee of the Montpellier University Hospitals (n° 2008-03-ESSS-V2 and n°2009-04-BPCO-V2) and conducted in accordance with the Helsinki declaration and the European guidelines for “good clinical practice”.

**Anthropometric data**

Weight (W) and height (H) were measured, and the body mass index (BMI) was calculated (BMI = W/H^2^). Fat-free mass (FFM) was estimated using a multifrequency bioelectrical impedancemetry (BIA) (QuadScan 4000, Bodystat, Isle of man, UK) [S1], and the fat-free mass index (FFMI) was calculated (FFMI=FFM/H^2^).

**Pulmonary function tests**

All subjects underwent spirometry (Transmural Bodybox 2800; Sensomedics, Yorba Linda, CA). FEV_1_ (forced expiratory volume in 1 second) and FVC (forced vital capacity) were measured. The FEV_1_/FVC values were compared with normal values [S2].

**Exercise testing and peripheral muscle function assessment**

A 6-minute walking test, which is routinely used by our group, was performed in a 30-meter corridor. The 6-minute walking distance was compared with reference values [S3]. The quadriceps muscle voluntary contraction was assessed with the usual methods of our group [S4,S5].

All subjects performed the pulmonary and muscle function tests at INSERM U-1046, CHRU Montpellier, France, or at the “La Solane” and “La Vallonie” Pulmonary Rehabilitation Centers in Osseja and Lodève, France, respectively.

**Immunostaining and fluorescence microscopy**

Myoblasts and myotubes were fixed in 3.7% formaldehyde in PBS followed by a 5-min permeabilization in 0.1% Triton X-100 in PBS. Immunostaining was performed using various specific antibodies and Hoechst 33258 for DNA staining. Images were captured with an AxioCam MRm CCD camera (Carl Zeiss, Oberkochen, Germany) driven by AxioVision 4 software (Carl Zeiss) on a Zeiss AxioImager M1 microscope (Carl Zeiss).

**SDS-PAGE and Western immunobloting**

Cells were lysed in hypotonic buffer (20 mM Tris pH7.5/10 mM NaCl/1 mM DTT/protease inhibitor cocktail; Sigma-Aldrich, St. Louis, MO) and protein concentration was determined using Bio-Rad Protein Assay (Bio-Rad Laboratories, Hercules, CA). 20 to 50 μg of proteins were separated by SDS-PAGE and transferred to PROTRAN nitrocellulose (Whatman, Dassel, Germany) or Immobilon-P PVDF (Millipore, Bedford, MA) membranes. Proteins of interest were revealed by specific antibodies, followed by enhanced chemiluminescence. Scanned radiographs were quantified using ImageJ software (National Institutes of Health, Bethesda, MD).

**Myoblast and myotube characterization**

Myoblasts, seeded at 50,000 cells/dish onto 35-mm collagen-coated dishes, were grown in proliferation medium for 72 hours. Cells were then harvested and counted (*x* cells), and the doubling time in hours was calculated as: 72/((ln*x*/50,000)/ln2).

The proliferation capacity was calculated after immunostaining with an anti-Ki67 antibody, an anti-desmin antibody and Hoechst 33258, as the ratio between the number of Ki67-positive nuclei and the total number of nuclei in a given field. 300 to 500 nuclei were counted per culture in at least 6 different fields.

The myogenic fusion index was calculated after immunostaining with an anti-troponin T antibody and Hoechst 33258, as the ratio between the number of nuclei into multinucleated myotubes and the total number of nuclei in a given field. 400 to 700 nuclei were counted per culture in at least 6 different fields.

The myotube diameter was evaluated after immunostaining with an anti-troponin T antibody and Hoechst 33258. Using ImageJ software, 3 measurements of the diameter were taken along each myotube, in at least 6 different fields, for at least 100 myotubes per culture.

**Oxidative stress assessment**

The OxyBlot Protein Oxidation Detection Kit (Millipore) was used to detect carbonyl group formation into protein side chains. Briefly, 15 μg of protein lysates extracted from myoblasts or myotubes were derivatized to 2,4-dinitrophenylhydrazone (DNP-hydrazone) by reacting with 2,4-dinitrophenylhydrazine solution for 15 minutes. Then, 7.5 μl of neutralization solution with 2-mercaptoethanol were added to the sample mixture. The derivatized samples were separated by SDS-PAGE and transferred to nitrocellulose membrane, and a rabbit anti-DNP primary antibody was used to detect total protein carbonylation.

HNE is a stable product of lipid peroxidation. HNE-modified proteins were detected by Western immunoblotting. Briefly, 50 μg of proteins from myotube lysates were separated by SDS-PAGE and transferred to PVDF membrane. Membranes were then probed with a primary anti-HNE antibody (Abcam, Cambridge, UK) for assessment of total lipid peroxidation.

For each polyacrylamide gel, a protein lysate from a patient was loaded in the first lane as an internal control, for normalization of the detected signals for each pair of gels. These control lanes were deleted from the autoradiographs before assembling the figures, for easier visualization of the results.

**H_2_O_2_-induced oxidative stress**

Myoblasts cultured in proliferation medium were incubated with an increasing concentration of H_2_O_2_ (100 to 900 μM) for 18 hours. Total cells were harvested and the mortality rate was assessed for each myoblast culture, for each H_2_O_2_ concentration, using Trypan blue staining.

**Antibodies and reagents**

Mouse monoclonal anti-desmin, anti-troponin T, anti-α-tubulin, anti-MHC1, anti-MHC2 and rabbit polyclonal anti-Ki67 were purchased from Sigma-Aldrich; rabbit polyclonal anti-catalase (H-300) and anti-myogenin (M-225) from Santa Cruz Biotechnology (Santa Cruz, CA); rabbit polyclonal anti-Mn SOD and anti-Cu/Zn SOD from Stressgen (Enzo Life Sciences, Farmingdale, NY); rabbit monoclonal anti-GPx1 from Epitomics (Burlingame, CA); rabbit polyclonal anti-dystrophin from Millipore (Bedford, MA); rabbit polyclonal anti-GAPDH from Abcam (Cambridge, UK); rabbit monoclonal anti-ERK1/2, anti-phospho-ERK1/2, anti-COX IV, anti-phospho-AKT and rabbit polyclonal anti-AKT from Cell Signaling Technology (Danvers, MA); rabbit polyclonal anti-MurF1 from GeneTex (Irvine, CA). Hoechst 33258 was purchased from Sigma-Aldrich.

**qPCR and primers**

Total RNA was extracted from myoblast and myotube cultures using TRIzol Reagent (Fisher Scientific, Pittsburgh, PA). First strand cDNA was synthesized from 1μg of total RNA using the Verso cDNA Synthesis Kit (Fisher Scientific). qPCR was performed in duplicate wells as follow: 40 cycles of amplification with 10 s at 95°C, 20 s at 60°C and 20 s at 72°C. After amplification, the melting curve was assessed to ensure the presence of a single product. The qPCR experiments were repeated twice with the LightCycler 480 system (Roche Applied Science) and SYBR Green 1 Master Mix (Roche Applied Science). Ct values of the target gene were normalized to Ct values of the house-keeping gene GAPDH, and pooled RNA were used as a calibrator. The expression level of each transcript was determined using the 2-ΔΔCt method.

Primers used are:

MyoD:

forward primer ACAACGGACGACTTCTATGAC ;

reverse primer TGCTCTTCGGGTTTCAGGA

Myf5:

forward primer CATGCCCGAATGTAACAGTC ;

reverse primer CCCAGGTTGCTCTGAGG

myogenin:

forward primer ACCCCGTTCTATGATGG ;

reverse primer ACACCGACTTCCTCTTACACA

IGF-1:

forward primer TGAGCTGGTGGATGCTGTTGAGTT ;

reverse primer TGCACTCCCTCTACTTGCGTTCTT

PGC-1α:

forward primer TGCTAAACGACTCCGAGAAC ;

reverse primer GCCATCAAGAAAGGACAGATAATC

MurF1:

forward primer AAA CAG GAG TGC TCC AGT CGG ;

reverse primer CGC CAC CAG CAT GGA GAT ACA

atrogin-1:

forward primer CGA CCT CAG CAG TTA CTG CAA ;

reverse primer TTT GCT ATC AGC TCC AAC AG

Nedd4:

forward primer ACCACAACTTGGACTCGACC ;

reverse primer GTCTTTCACAGCCCGACGTA

myostatin:

forward primer GAGCATTGATGTGAAGACAGTG ;

reverse primer GTTACCTTGACCTCTAAAAACGG

FoxO1:

forward primer TTTGCGCCACCAAACACCAGTT ;

reverse primer TGGCTGCCATAGGTTGACATGA

FoxO3:

forward primer ACGTGATGCTTCGCAATGATCCGA ;

reverse primer ACTCAAGCCCATGTTGCTGACA

**References**

S1. **Kyle UG, Genton L, Karsegard L, Slosman DO, Pichard C.** Single prediction equation for bioelectrical impedance analysis in adults aged 20--94 years. Nutrition. 2001 Mar;17(3):248-53.

S2. **Quanjer PH, Tammeling GJ, Cotes JE, Pedersen OF, Peslin R, Yernault JC.** Lung volumes and forced ventilatory flows. Report Working Party Standardization of Lung Function Tests, European Community for Steel and Coal. Official Statement of the European Respiratory Society. Eur Respir J Suppl. 1993 Mar;16:5-40.

S3. ATS statement: guidelines for the six-minute walk test. Am J Respir Crit Care Med. 2002 Jul 1;166(1):111-7.

S4. **Gouzi F, Prefaut C, Abdellaoui A, et al.** Evidence of an early physical activity reduction in chronic obstructive pulmonary disease patients. Arch Phys Med Rehabil. 2011 Oct;92(10):1611-7 e2.

S5. **Koechlin C, Couillard A, Simar D, et al.** Does oxidative stress alter quadriceps endurance in chronic obstructive pulmonary disease? Am J Respir Crit Care Med. 2004 May 1;169(9):1022-7.

**Figure legends**

**Fig. S1:** Expression levels of myogenesis markers. MyoD mRNA expression in myoblasts (**A**) and myotubes (**B**). Myf5 mRNA expression in myoblasts (**C**) and myotubes (**D**). Myogenin mRNA expression in myoblasts (**E**) and myotubes (**F**). Data are normalized to GAPDH expression. (**G**) The mean values from the quantification of two Western blots assessing myogenin expression in cultured myoblasts at 2 days of differentiation, relative to tubulin expression. Cells are derived from 8 control subjects (CONT) and 8 COPD patients (COPD). Values are represented in arbitrary units (a.u.).

**Fig. S2:** (**A**, **B**) Statistical analysis of patients in the COPD group showing correlations between the cultured myotube diameter (Myotube diameter) and: (**A**) the quadriceps fiber cross-sectional area (Fiber CSA) and (**B**) the quadriceps maximal voluntary contraction (MVC). Data for some subjects are not presented due to unavailable values for their fiber CSA and MVC.
